# Post-Partum Hemorrhage

**Published:** 1892-06

**Authors:** Roger Williams

**Affiliations:** Pittsburg, Pa.


### POST-PARTUM HEMORRHAGE.*

BY
ROGER WILLIAMS, M. D.,
PITTSBURG, PA.

There is no time when action is more promptly required, and knowledge and wisdom brought into play, than, when alone, one

*Read before the Medical Society of Allegheny county, Pa., Feb. 16, 1892.

is brought into the presence of a post-partum hemorrhage. There is no emergency in the physician's life that gives less time for consultation and reflection, and no time more exacting to do the right thing, at the right time and in the right way. Post-partum hemorrhage is one of the most frequent complications of delivery. Call to mind, if you please, the many conditions you guard against in a labor about which you have doubt, and, in a vast majority, post-partum hemorrhage is first to be feared. There is something more than intuition that places one on guard, for in the vast majority of subjects to this complication we find indisputable indexes pointing to the subject in question. The patient, by reason of our wrongly-directed civilization and her whole surroundings, has engrafted a lax habit of body, inviting uterine inertia, and as a consequence, post-partum hemorrhage. We find illustrations of this in the upper ranks of life, for they are most prone by reason of the demands of society.

Among the causes contributing may be mentioned the site and size of the placental attachment. A placenta attached to the fundus may have slight uterine attachment, and the uterine sinuses closed by nature's processes. Where the placenta is thin and covers a great surface, we may have ectasis of the uterine vessels, and as a result, hemorrhage. Local œdema, induced by perverted secretion and inflammation, may prevent contraction, and as a result we have immediate hemorrhage. Complication to the funis, where it is interfered with and shortened, may cause a too rapid placental displacement, and therefore profuse hemorrhage even before the child is extruded from the maternal parts.

Retained portions of placenta is a frequent cause of post-partum hemorrhage. I call to mind a case that occurred in my practice during my residence in Philadelphia. Mrs. S., four and a half months in the family way, was pushed off the pavement by a swinging gate, and fell in a sitting posture to the gutter. She promptly aborted, and as the placenta could not immediately be taken away, she suffered profuse hemorrhage which lasted until the placenta was expelled.

There may be a proliferation of the placental vessels constituting an extra or supplementary lobe, and the retention of this even after the placenta is removed will cause profuse hemorrhage.

Plural births, with double placenta, is not an unfrequent cause of post-partum hemorrhage, for at times the uterus seems to be irresponsive to all our efforts, and a hemorrhage beyond what we desired is the result. I call to mind a case in illustration : Mrs. G. was delivered of twins, and the delayed second delivery was caused by an inertia induced by a beginning cancerous growth. The last delivered child was in part covered by a fleshy mass not unlike placenta, and was born dead.

Irregular contraction is a cause of post-partum hemorrhage not uncommon. Part of the muscular fibres are relaxed, whilst part are in a state of contraction; the former often over the placental site, and is the cause of considerable hemorrhage. By palpation this condition is easily discerned.

Hour glass contraction is also a cause of post-partum hemorrhage; rare it is true, yet nevertheless occurring. This condition follows often the obstetrician, who, by efforts of traction, excites uterine contraction at the seat of irritation—often the placental attachment, and many times the internal os, and, as a cause, we have a diaphragm temporarily placed, with placenta in part or wholly included within the uterus, and hemorrhage of greater or less extent resulting. This condition rarely, if ever, follows after efforts of placental expression, and as this form of delivery of the after-birth grows into favor, hour glass contraction grows less.

Encystment of the placenta is one of the rare complications of labor, and when it occurs, the placental site remains more or less paralyzed, whilst the remaining muscular fibres are in contractility. This form of trouble in the delivery of the after-birth is apt to be mistaken for adherent placenta, and oftentimes a part of it is left intra-uterine, as a result of wrongly directed effort, and hemorrhage results, complicated at times by sepsis.

The placenta and membranes, and also the decidua, may be the seat of calcareous or fibroid degeneration, or the decidua may be abnormally thick. When an accident of this character to a part of the placenta or its membranes occurs, we have a cause of irritation not unlike a foreign body placed against the maternal tissues, and as a consequence, loosening of attachment in part, or an inflammation that causes adherent placenta. I think the many cases of adherent after-birth can be traced to one of the conditions mentioned; but, be this as it may, the hemorrhage in this complication is not to be lightly considered, for the placental site is in a state of irritation, and its muscular fibres will respond freely.

Laceration of the cervix is a cause of post-partum hemorrhage not unfrequently. At times the structures of the uterine neck are hard and brittle, as well as unyielding, and, during labor, are apt to suffer to a greater or less extent. Mrs. W., a patient of mine, was delivered during my attendance at the A. M. A., at Nashville, and suffered an extensive laceration of the cervix, which was the immediate cause of an extensive hemorrhage controlled by styptic injections and plugging.

Lacerations in the vulva are not unfrequently the cause of violent and prolonged hemorrhage. Mrs. D., primipara, was delivered by me in December last, and there was a band of tissue broad as a common lead pencil joining the labia minora, which had escaped my examination, and when labor was completed the torn end of this small impediment caused quite a hemorrhage, which was, with difficulty, controlled by application of Monsell salt.

Growths within the uterus, even when too small to be detected during labor, are oftentimes the cause of alarming post-partum hemorrhage. I saw a case of this character with Dr. McNeil, the history of which he has kindly furnished. Mrs. K., a German, aged thirty-eight, confined December 16, labor lasting twenty-four hours, antero-posterior diameter narrow, delivery with instruments, and nothing marked the labor out of the usual order until the eleventh day, when violent hemorrhage occurred, keeping up until the patient was almost exsanguinous. Prompt injection of hot water with vinegar, and teaspoonful doses of ergot relieved the hemorrhage and expelled a small fibroid which was the cause of the hemorrhage. All went well until January 3d, when during one of the quick atmospheric changes, the patient had a chill; rise of temperature, and died with pnuemonic congestion January 5th.

The character of a contraction has much to do with post-partum hemorrhage. Admit we have a perfect contraction immediately after labor, if it is not permanent, and tonic, the relaxation may induce a fearful hemorrhage. Post-partum hemorrhage and permanent contraction are incompatible I will admit, yet on examination one hour after labor and finding the uterus soft and flabby, and where coagulæ have not formed in the uterine sinuses, hemorrhage will surely result. Nature has her own way of controlling hemorrhage by contractility of the uterus, and by plugging of the orifices of the uterine sinuses; and to assist by art when nature fails, is not an ignoble part the physician plays in attendance upon labor.

Not long ago a physician left the bedside of a patient with violent hemorrhage, with hurried direction to those who could not do what he should have done, in a fruitless quest for an instrument for transfusion. Had he gone for wings in order that she might rise into the circumambient clouds, he should have been held less censurable, for when he returned life and hemorrhage both had ended.

I need not speak of the secondary causes of hemorrhage at great length, for that man who is not awake to the importance of the uterus worn out in a prolonged and exhausting effort in expelling the fœtus, or the uterus over-distended by excessive amounts of liquor amnii, or the consequences of a multiple birth, should not practice the obstetric art. Rapid emptying of the uterus is—excepting when filled with clots as a consequence of uterine hemorrhage—baneful, jeopardizing and unskillful, and to do this to the over-distended uterus is criminal. The walls of an over-distended uterus are to all intents and purposes paralyzed walls, and when the shock comes from relief of distension, it is the rule to expect retardation in contraction, and as a result, hemorrhage of a greater or less extent.

Concealed hemorrhage, following delivery, is a subject of interest to all. That fatal hemorrhage of this character exists many here will attest, and the obstetrician should ever be on guard. The flabby uterus rendered insensible, and worn out by long continued labor, may by bleeding within itself rob its subject of life. The external evidences of hemorrhages may be normal, but where there is evidence of hemorrhage the practiced eye detects at once. The conditions leading up to this are referable to clots, and at times membranes act as a valve within the internal os. Illustrative of this, I am furnished the history of Mrs. ——, delivered at Homewood, in the summer of 1891, who passed through a normal labor until the ninth day, when her physician was sent for, and found her pale, weak and bloodless. A syringe and hot water removed two quarts of clots. Phlebitis complicated the case, but the patient recovered.

Patients constitutionally predisposed to flooding are perhaps the most interesting of the class mentioned, and well merit the appellation of "bleeders." They follow the inclination of predisposition, in spite of every effort the physician puts forth, to a certain extent, and at times only mechanical pressure will relieve.

There are few sights more dreadful to look upon than the worst cases of post-partum hemorrhage, A reign of terror ushers it into view, and that one who is at his post, equal to the occasion and to the emergencies that arise, has within him the elements of a hero. The pulse is a mere thread, or perhaps is imperceptible ; syncope manifests itself, and with it is born a hope of thrombosis in the venous sinuses; and yet there may be fatal syncope. Intense weariness and faintness come on, and the patient, wildly tossing her arms, is with difficulty restrained. Respiration becomes gasping and sighing, suffocation seems impending, and the patient calls for more air. The skin is deadly cold, the face is pale and covered with profuse perspiration. If the hemorrhage is not controlled, loss of vision, jactitation, convulsions and death speedily follow.

The treatment of post-partum hemorrhage, principally, should consist in using preventive measures. If the uterus is firmly held in contraction until the secundines are extruded, and the fundus held in the grasp of the hand until the first half hour is passed; if only, when assured that permanent contraction has manifested itself, the binder is firmly applied, and, if a full dose of ergot is immediately given, cases of post-partum hemorrhage would be much less frequent. If the history of the patient is one of post-partum hemorrhage, then a greater care should be taken. It will be well to administer subcutaneously, a half-hour before the supposed termination of labor, a full dose of ergotine. If then the os is dilatable, and one has prepared for the emergencies that *might* arise, rupture the membranes, and take advantage of every means to insure regular and, if possible, permanent contraction. If the pulse does not fall slightly below, or comes to the normal in twenty minutes after labor is completed, this in itself is indicative of impending hemorrhage, and then prompt treatment of tendencies, especially referable to relaxation, demands the intra-uterine administration of remedies to control hemorrhage. Ice, with uneven edges removed by immersion in hot water, carried to the fundus, and held until contraction is induced, or ice-water—if the surrounding parts are protected—by injection, or water of the temperature of 120 to 130 degrees, by the same means, and persisted in until contractions are invoked. Absolutely hot water favors coagulation in the uterine sinuses, and in those nervously disposed is a form of treatment suited. It is supposed that subcutaneous injection of ergotine has been used, or that ergot in the form of fluid extract has been given in sufficient quantities. Antiseptic wool carried to the bleeding surfaces is to be used, and in the selection of remedies, there is the choice of dilute acetic acid, chloroform or the mineral astringents. If a powder-blower is at hand, and the parts admit, the use of pulverized nutgalls will serve a goodly purpose, and is a safe means of arresting hemorrhage. The application of tr. iodine is also efficacious, and can be readily applied on wool. Strength of the patient must be maintained, compensation for the loss of blood looked after, and to this end, injections of drachm doses of ether plays a part, with the inhalation of nitrite of amyl, or the injection of stimulants. Alcoholic-saline intravenous injection gives back tension and supplies, in part, the fearful waste. These failing, the abdominal aorta can be compressed, or the uterus held firmly by the hand sufficiently long to aid in the formation of coagulæ.

